# Extract from the Address of Dr. George Wigg

**Published:** 1889-06

**Authors:** 


### EXTRACT FROM THE ADDRESS OF DR. GEORGE WIGG

### Before the Annual Meeting of Homœopathic Medical Society, held in Portland, May 14th and 15th.

*Fellows of the Homœopathic Medical Society of the State of Oregon:*—The days of another year of anxiety and toil have thrown their lengthened shadows athwart our pathway. Another twelve months have been from the old bell of time tolled into eternity. How swiftly have the sands run, and how rapidly the year has drawn to its close. It is gone; and on its pages are inscribed our every act; acts that are now beyond our control to alter or amend, however much we might wish, not one line or syllable can we erase, for, as time past never returns, so an act once accomplished, no matter whether for good or evil, is done for all time.

The mighty waves of ages may continue to wash against the shores of the past, and throw oblivion over its pages, but are powerless to efface a single letter.

To-day brings us face to face with another milestone, and the inscription upon it informs us that the Homœopathic Medical Society of the State of Oregon has arrived at the thirteenth year of its existence. And on this anniversary day, we, the members forming this society, have assembled. I trust the object of our thus meeting is for the purpose of exchanging fraternal greeting, extending our acquaintance, and comparing notes upon the results of our experience in the art of healing, for the benefit of humanity.

We have met, not only to receive, but also to give, and in so doing we must not lose sight of the fact that a one talent may be to this society, at this time, of as much importance as a ten. No doubt, ladies and gentlemen, that, during the past year, you have met with both success and disappointment. Let the former inspire you with hope and confidence, and the latter urge you to investigate and improve. Again, some of you may have been compelled to bear heavy burdens and undertake great responsibilities alone, when you would have gladly shared them with a professional brother, but, owing to unkindness, you have been left alone with your patient, to battle with disease as best you could. But, thanks to the true spirit of inductive philosophy, found in Dr. Samuel Hahnemann, you have, by keeping your eyes fixed upon the pole star, *Similia similibus curantur*, passed through the trying moments, bringing your patients back to health, and their friends to a haven of joy.

A careful captain will often take his soundings, examine his chart, and note the needle in his compass. This he does to guard against danger, and that he may bring safe to land the charge intrusted to his care. Thus should we, as physicians, make this an occasion for observation and ascertain the condition of Homœopathy generally. As homœopathists, we must bear in mind that we are not like those brought up at the feet of Hippocrates, cast adrift on the great ocean of human affliction, with no pole star or beacon light to guide them to a haven of safety. They find themselves tossed in the surging sea of suffering humanity without compass or sheet anchor. They hear the groans and cries of a whole world of men, women, and children bowed down beneath the curse of disease, and as they watch the craft as it drifts into the whirlpool of death they exclaim, in the language of Dr. Hufeland, "that more harm than good is done by physicians, and I am convinced that had I left my patients to nature, instead of prescribing drugs, more would have been saved." It was the opinion of Sir John Forbes that in a "considerable proportion of diseases it would fare as well, or better, with patients, in the actual condition of the medical art as more generally practiced, if all remedies, at least all active remedies, especially all drugs, were abandoned." And Dr. Good says it is his experience that "the science of medicine is a barbarous jargon, and the effect of their medicine on the human system was, in the highest degree, uncertain, except, indeed, that they had already destroyed more lives than war, pestilence, and famine combined."

These are wails from the very deeps of the souls of men who have not willingly drifted away from what was taught them in their college days, and launched out on an ocean of doubts and treacherous snags.

At the trial of Jesus Christ, the Jews exclaimed, "We have a law, and by our laws He ought to die." We, as homœopathic physicians, cry to every afflicted son and daughter of a fallen race, "We have a law, and by our law you ought to live." For ours is a law by which every curable disease can be cured. Thousands live to-day, standing monuments to the truth of this assertion.

The application of this law of cure consists in the search for a homœopathic, specific remedy, by the comparison of the totality of the symptoms of the natural disease with the list of symptoms of our tested drugs, among which a morbific potency is to be found. And it is necessary in making this comparison, the more prominent, uncommon, and peculiar characteristic features of the case are especially, and almost exclusively considered and noted, for these in particular should bear the closest similitude to the symptoms of the desired medicine that is to accomplish the cure. This is the homœopathic law or art of healing, of which Hahnemann says, "Is the only correct, the only direct, and the only possible means to be employed by human skill, as surely as it is possible to draw but one straight line between two given points." And we say to those who are antagonistic to this truth, you may as well try to control the blast of the hurricane, or the ebb and flow of the tide, as to try and control or prevent the onward march of this wonderful law of cure.

You may bind its discoverer in shackles never so strong, shut him up in prison with walls as thick as masons can build them, and cells as dark as Egyptian night, but no sooner is he brought out into God's free sunlight, than he exclaims: "*Similia similibus curantur.*" And this "*similia*" is your beacon light, your sure guide in the discharge of your professional duties. This star came into life in Meissen, in the kingdom of Saxony, on the 10th of April, 1755, and year after year it has been growing brighter and brighter, and to-day its light shines over the whole civilized world. It will continue to shine, and its golden beams shall lengthen, and its halo of truth increase until it is lost in the effulgent glory of that Great Physician who dwells in that land "where there shall be no more death."

It will no doubt be gratifying to you all to learn that the past year has been the most prosperous one our school has ever experienced. The march of Homœopathy has been forward, every step taken has been a gigantic one. At no time in its history has the star of Hahnemann shone so brightly as it shines to-day, and its brightness is attracting the attention of the thoughtful in every department of medical science. One by one its professors are adopting the principles taught in the *Organon of the Art of Healing.* I venture the assertion that the day is not far distant when this mode of practice that our school has for the last century been contending for will be recognized as the only system of medical practice worthy the name.

Already it has deprived the prescription of "ye olden times" of its head and tail. And to-day we find the chemist and pharmacist vying with each other in the preparation of the smallest doses outside the homœopathic school, thereby trying to help the old school boys imitate the homœopaths in a crude and bungling manner. The time predicted by Hahnemann has already come. His system has indeed grown into an oak of God, which the winds and storms of our adversary can no longer shake. Its branches spread into all regions, and underneath them you may find the high and low, rich and poor, young and old drinking in its healing virtues which are being dispensed night and day by an army of twenty thousand physicians, while its laymen are counted by the millions.

In this mighty army we may see the leaven at work, bringing to the surface new thought and grand developments, and the working will go on until upon the law *similia similibus curantur* stands the grandest science of medicine the world will ever look upon.

Opposition we must expect. A kite will never rise with the wind, but against it; even a head wind is better than none. As homœopathic physicians we must not expect to work our passage in a dead calm. I think it would do us all good to get a few such knocks and rub-downs as Hanhemann and his early followers received while at work sowing the seed which now yield the fruit from which we draw our supply.

